# A graphene quantum dots–glassy carbon electrode-based electrochemical sensor for monitoring malathion

**DOI:** 10.3762/bjnano.14.56

**Published:** 2023-06-09

**Authors:** Sanju Tanwar, Aditi Sharma, Dhirendra Mathur

**Affiliations:** 1 Centre of Nanotechnology, Rajasthan Technical University, Kota, Rajasthan, India; 2 Materials Research Centre, Malaviya National Institute of Technology, Jaipur, Rajasthan, India

**Keywords:** cyclic voltammetry, differential pulse voltammetry, electrochemical impedance spectroscopy, electrochemical sensor, graphene quantum dots, malathion

## Abstract

Graphene quantum dots (GQDs) were made via a hydrothermal process with glucose as a precursor undergoing carbonization. Different spectroscopic techniques were used to analyze the optical characteristics of GQDs, including UV–visible, photoluminescence, FTIR, and Raman spectroscopy. Atomic force microscopy, transmission electron microscopy, and X-ray diffraction were used to characterize the morphological and structural properties of GQDs. An electrochemical sensor was developed by drop casting GQDs on a glassy carbon electrode (GCE). The sensor detects the organophosphate pesticide malathion in a selective and sensitive manner. Using cyclic voltammetry, the sensor’s oxidation–reduction behavior was investigated. Electrochemical impedance spectroscopy was conducted to study the electrochemical properties of the modified the GQDs/GCE working electrode, which showed excellent charge transfer properties. We measured malathion in varying concentrations between 1 to 30 µM using differential pulse voltammetry, which resulted in a limit of detection of 0.62 nM. GQDs can thus be used to develop electrochemical sensors for the detection of pesticides in water.

## Introduction

Global population growth makes food production more challenging, and pesticides are therefore used in agriculture in greater quantities than in the past to maintain and increase crop yields [1–2]. Pesticides containing organophosphates are widely used due to their availability as efficient, low-cost pesticides. It is important to recognize, however, that excessive pesticide use poses a negative impact on the environment and human health because of biomagnification and persistence [3]. One example of an organophosphate insecticide is malathion, which kills insects such as fleas and ants that attack plants. Malathion has been detected so far using chromatography [4–5], colorimetry [6], and mass spectrometry [7], although these methods are complicated and time-consuming and require expensive equipment with specialized expertise. It is therefore necessary to develop a technology that can detect pesticides quickly, easily, and economically.

With electrochemical detection techniques, a wide range of pesticides can be detected by the fabrication of simple, cost-effective, rapid, and high-throughput portable devices [8]. The application of electrochemical methods in detection of pesticides has already been extensively studied [9–13]. Nanomaterials are ideal for electrochemical sensing because of their unique properties such as high chemical stability, thermal conductivity, electrical conductivity, and large surface-area to volume ratio to provide enhanced analyte interaction with the sensing surface [14]. Carbon-based nanomaterials and nanocomposites are being investigated for the electrochemical detection of a variety of pesticides, including organophosphates, organochlorines, and carbamates [15–17]. The use of graphene and its derivatives is widespread for electrochemical detection since 2D graphene sheets provide numerous electrochemical sites for the detection of target molecules, while electrons in the sp^2^-hybridized p_z_ orbital have a faster electron transfer rate, which enhances response time and lowers the detection limit [18].

In an effort to combine the properties of carbon dots and graphene, graphene quantum dots (GQDs) with a size smaller than 100 nm and only a few layers of graphene (3 to 10 layers) have been developed as a new class of carbon nanomaterials [19]. Scientists have explored the possibilities of developing sensing devices based on graphene quantum dots in recent years [20–23]. In 2015, Dong et al. prepared an oxime-based sensor via attaching pralidoxime on a GQDs-modified GCE for detecting the organophosphorus pesticide fenthion [24]. In 2018, Sahub et al. worked on a biosensor platform consisting of graphene quantum dots functionalized with acetylcholinesterase and choline oxidase for the detection of the organophosphate pesticide dichlorvos [25]. In 2018, Qian Liu et al. developed a photo-electrochemical sensor with nitrogen-functionalized graphene quantum dots and 3D bismuth oxyiodine hybrid hollow microspheres for the detection of chlopyrifos [26]. In 2020, Jiménez-López et al. worked on a fluorescent probe containing graphene quantum dots and silver nanoparticles for glyphosate detection [27]. In 2021, Xu Dan et al. developed a histidine-functionalized nickel/silver/graphene quantum dot/graphene hybrid for the colorimetric detection of malathion [28].

This paper describes the development of an electrochemical sensor based on a graphene quantum dot-modified glassy carbon electrode (GQDs/GCE) for the determination and quantification of the organophosphate pesticide malathion. Graphene quantum dots were synthesized hydrothermally using glucose as precursor. The glassy carbon electrode that served as working electrode in the electrochemical cell was modified with graphene quantum dots by drop casting. To evaluate the modified electrode’s oxidation/reduction behavior and charge transfer resistance, cyclic voltammetry and electrochemical impedance spectroscopy were performed. An investigation of the relationship between concentrations and peak currents was conducted using differential pulse voltammetry (DPV). In this study, the modified GQD electrodes were found to be effective sensing platforms for the electrochemical detection of organophosphate pesticides, particularly malathion.

## Experimental

### Materials

Glucose (C_6_H_12_O_6_), conc. sulfuric acid (98% H_2_SO_4_), potassium hexacyanoferrate(III) (C_6_FeK_3_N_6_), and potassium chloride (KCl) were obtained from Fisher chemicals. Malathion (C_10_H_19_O_6_PS_2_) was obtained from Insecticides India Limited. Disodium phosphate (Na_2_HPO_4_·H_2_O), monosodium phosphate (NaH_2_PO_4_), sodium hydroxide (NaOH), ethanol (C_2_H_5_OH), and isopropyl alcohol (C_3_H_8_O) were procured from Rankem chemicals. Nafion (C_9_HF_17_O_5_S) and activated charcoal were taken from Fisher Scientific. For all experimental work and the preparation of stock solutions, deionized (DI) water was used.

### Synthesis of graphene quantum dots

Graphene quantum dots (GQDs) were synthesized using glucose as a precursor material via a hydrothermal route [29] with some modifications. Glucose (2 g) was dissolved in 20 mL DI water and filtered for the removal of undissolved particles through Whatman filter paper. In the above solution, 20 mL of conc. H_2_SO_4_ was added dropwise until it turned brownish under constant stirring. The hydrothermal treatment was conducted by heating the 40 mL suspension at 200 °C for 5 h in a 50 mL poly(tetrafluoroethylene)-lined autoclave. Washing with DI water was carried out in order to remove the acid from the resulting black suspension once it had been cooled to room temperature. NaOH solution was subsequently used to neutralize the solid collected after centrifugation. To obtain the GQDs, the final black suspension was filtered through a 0.22 µm syringe filter.

### Fabrication of the electrochemical sensor

**Figure 1:**
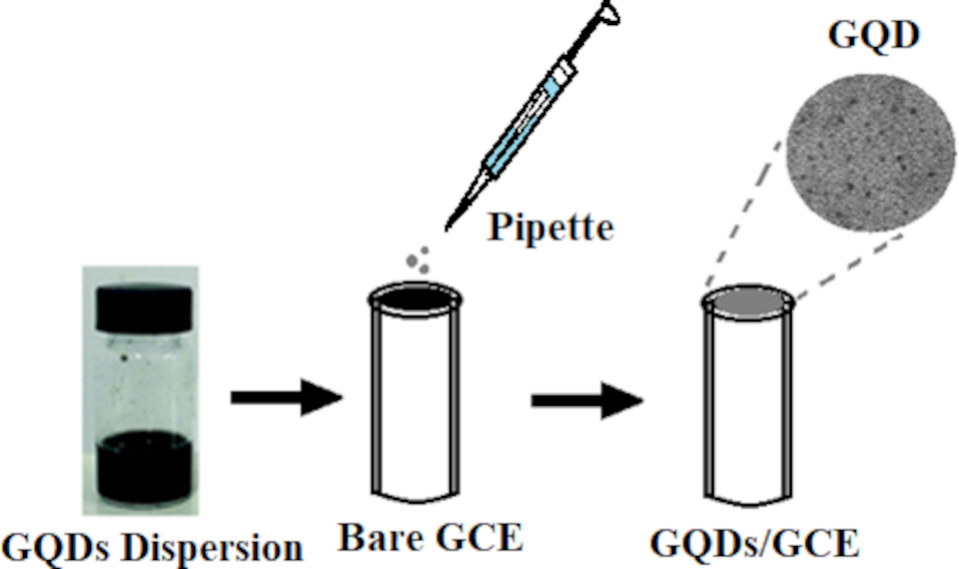
Fabrication of the GQDs/GCE electrochemical nanosensor for the detection of malathion.

A mirror-like surface was first achieved on the bare GCE by polishing it with 0.3 and 0.05 μm alumina powder. In the next step the GCE was sonicated in ethanol and rinsed with DI water to remove surface impurities. The GQDs-based ink was prepared in a glass vial with four components, that is 15 mg activated charcoal as a conductivity enhancer, 15 mg GQDs as modifying agent, 25 µL Nafion as binder, and 1 mL isopropyl alcohol as solvent. All components were sonicated for 30 min to create a homogeneous mixture that could be utilized for the modification of the bare GCE [29]. The final step was to drop cast 5 µL of the GQDs dispersion on the GCE surface and allow it to dry at room temperature. A GQDs-modified working electrode (GQDs/GCE) was obtained, which will be used as an electrochemical nanosensor in further studies for malathion detection (Figure 1).

### Characterization

FTIR, Raman, UV–vis, and fluorescence spectroscopy measurements were carried out to determine the optical properties of GQDs. A Perkin Elmer LAMBDA 750 spectrophotometer was used to record the UV–visible absorbance spectrum. The luminescence characteristics of the GQDs were investigated using a Perkin Elmer LS 55 fluorescence spectrometer. On a Perkin Elmer FT-IR Spectrum 2, FTIR spectra were measured in the range of 500–4000 cm^−1^ by making KBr pellets of the sample. At room temperature, an AIRIX STR 500 laser Raman spectrometer was used with Ar laser excitation at 532 nm. A Panalytical X-Pert Pro diffractometer with Cu Kα radiation (λ = 1.5418 Å) was used for investigating the structural properties of GQDs. Morphology and size of GQDs were confirmed with data obtained from a Bruker AFM analyzer atomic force microscope and a FEI Tecnai G2 20 S-TWIN transmission electron microscope.

### Electrochemical measurements

GQDs/GCE, Ag/AgCl, and a platinum wire were used as working, reference, and counter electrode, respectively, in all electrochemical experiments, conducted on a SP Biologic 150 electrochemical workstation at room temperature. A solution of 0.1 M KCl containing 0.05 M K_3_[Fe(CN)_6_] was used as an electrolyte to analyze the oxidation–reduction behavior of the working electrode through cyclic voltammetry. For detection and quantification of pesticides, differential pulse voltammetry experiments were conducted with 0.1 M phosphate-buffered saline at pH 7.

## Results and Discussion

### Characterization of graphene quantum dots

**Figure 2:**
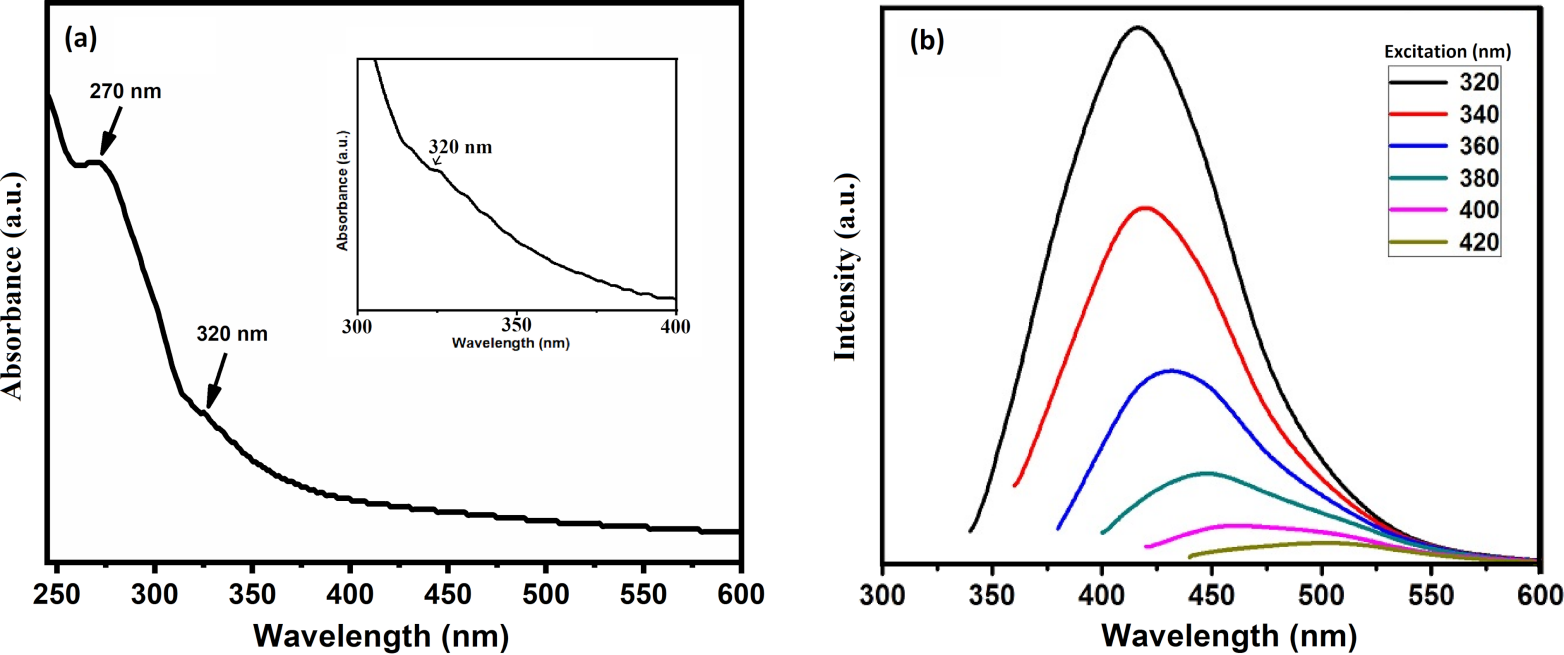
(a) UV–vis absorption spectrum and (b) photoluminescence spectra of GQDs.

The UV–vis absorption spectrum of the GQDs in distilled water is depicted in Figure 2a, which shows two prominent absorption peaks around 270 and 320 nm, in agreement with the data previously reported [30–31]. The shoulder at 270 nm is probably caused by π–π* transition of the C=C bonds, and the absorption hump at 320 nm is likely caused by n–π* transitions of the C=O bonds. As shown in Figure 2b, when the graphene quantum dot suspension was excited at 320 nm, the photoluminescence (PL) spectrum of GQDs showed a strong peak around 425 nm, similar to those reported for GQDs [32]. When excited at wavelengths between 320 and 420 nm, the PL peak shifts from 420 nm (violet) to 520 nm (green), and the PL intensity also decreases significantly. Therefore, it can be inferred that not only quantum size effects, but also defects on the surface, contribute to the PL in GQDs.

**Figure 3:**
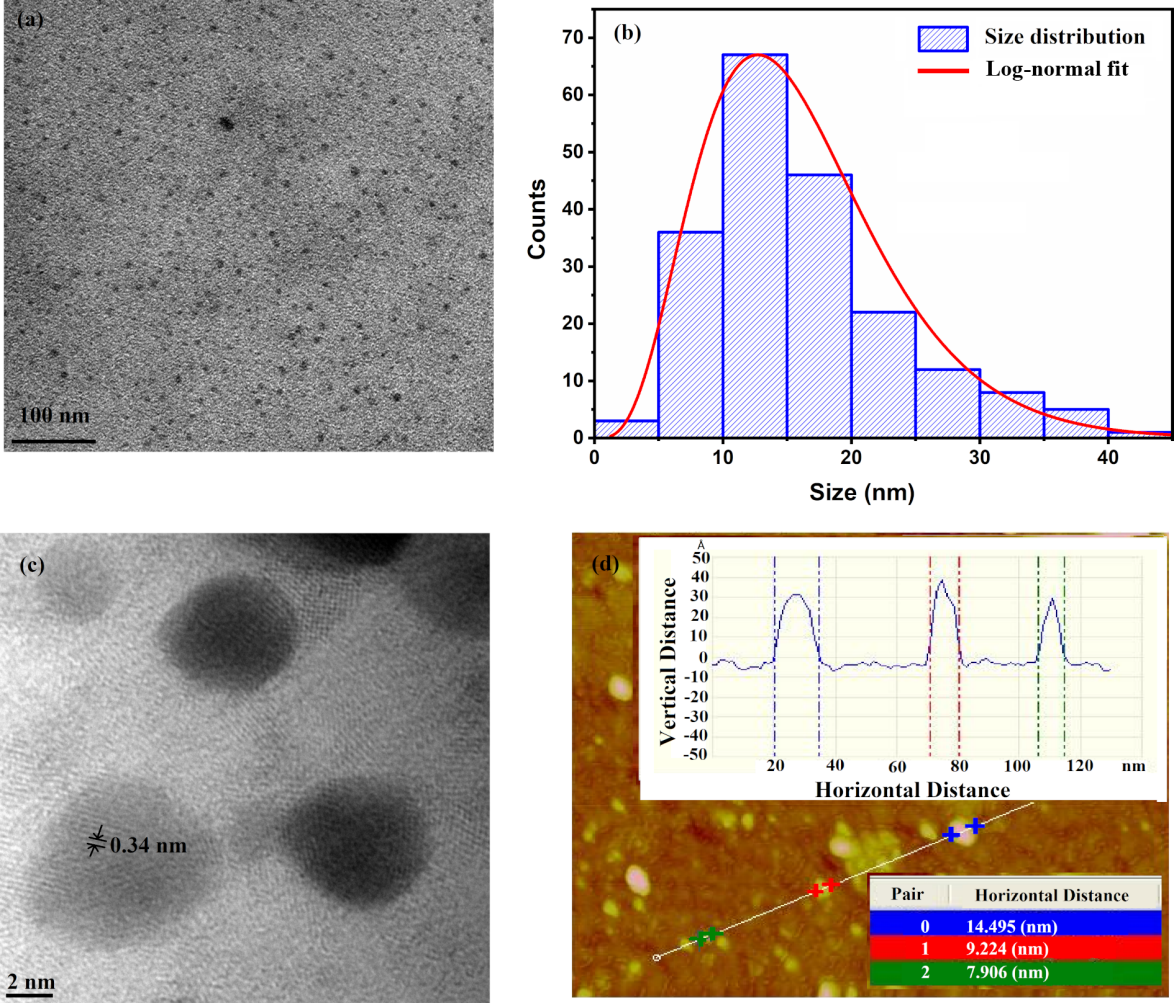
(a) TEM image, (b) size distribution along with log-normal fit, (c) HRTEM image, and (d) AFM image of GQDs.

Size and morphology of GQDs were characterized using TEM and AFM. The TEM micrographs shown in Figure 3a confirm the formation of evenly dispersed GQDs with almost spherical shape. Figure 3b shows the size distribution and the log-normal fit, from which a mean of 12.75 nm and a full width at half maximum (FWHM) of 15.41 nm were obtained. The GQDs vary in size from 5 to 40 nm, with the highest number of dots having a size in the 10–20 nm range. The HRTEM image of the GQDs in Figure 3c shows their crystalline structure. The lattice spacing obtained is 0.34 nm, which can be related to the (002) crystal planes of GQDs. Figure 3d shows an AFM image of the synthesized GQDs. The *x* axis and the *y* axis in the inset of the AFM image show the horizontal distance and vertical distance, respectively, covered by the GQDs. The variation in size of the GQDs can be determined from the *x* axis, while from the *y* axis, the thickness of the GQDs can be obtained. The average thickness of the GQDs is about 2.8 nm, which indicates the presence of 8–9 graphene layers, assuming an interlayer distance of 0.33 nm [33].

**Figure 4:**
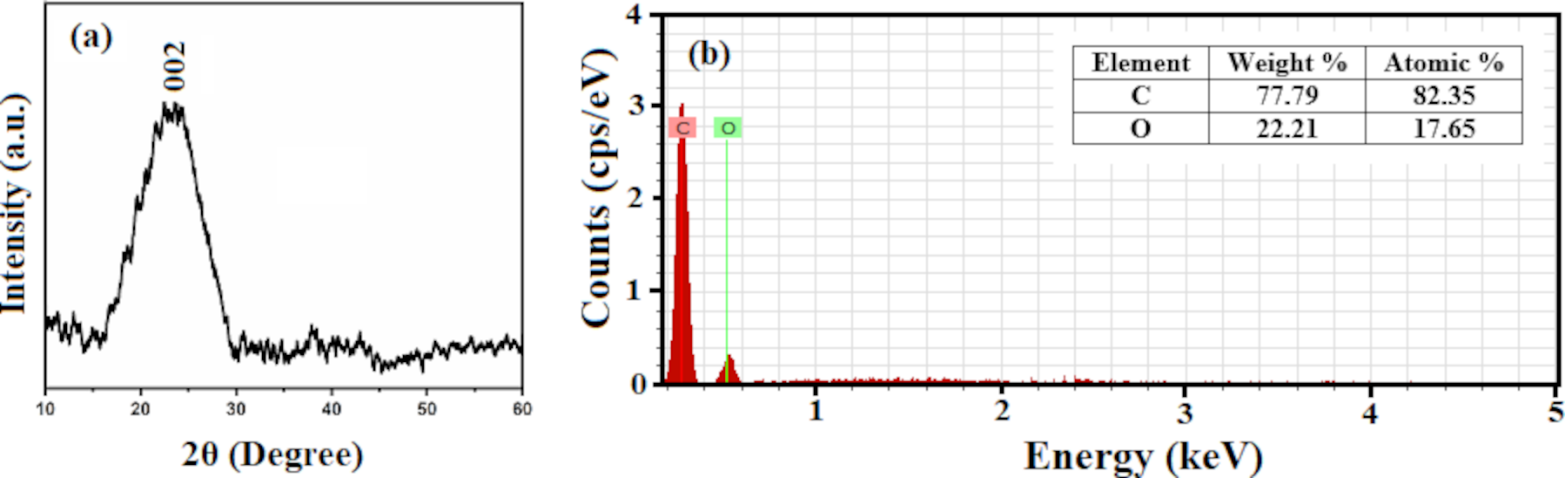
(a) XRD pattern and (b) EDX spectra (inset showing weight and atomic percent of carbon and oxygen) of GQDs.

The XRD pattern of the synthesized GQDs is shown in Figure 4a. A broad diffraction peak at 24.08° is obtained, which corresponds to the (002) crystal planes of the GQDs with a *d* spacing of 0.369 nm [34]. It can be inferred from the higher *d* spacing value of GQDs that oxygen containing functional groups are still present in GQDs even after hydrothermal treatment. Due to the nanoscale size of GQDs and a small number of graphene layers, the diffraction peak appears broad [35]. Using the FWHM of the diffraction peak, an average crystallite size of 2.69 nm was calculated for the synthesized GQDs using the Debye–Scherrer formula, *D* = 0.9λ/(β·cos θ), where *D* is the average crystallite size of the synthesized GQDs, λ is the X-ray wavelength, θ is the Bragg diffraction angle, and β is the FWHM. The elemental analysis of GQDs from EDX measurements is shown in Figure 4b. The EDX spectrum shows the presence of only carbon and oxygen in the GQDs with 82.35 atom % carbon and 17.65 atom % oxygen.

**Figure 5:**
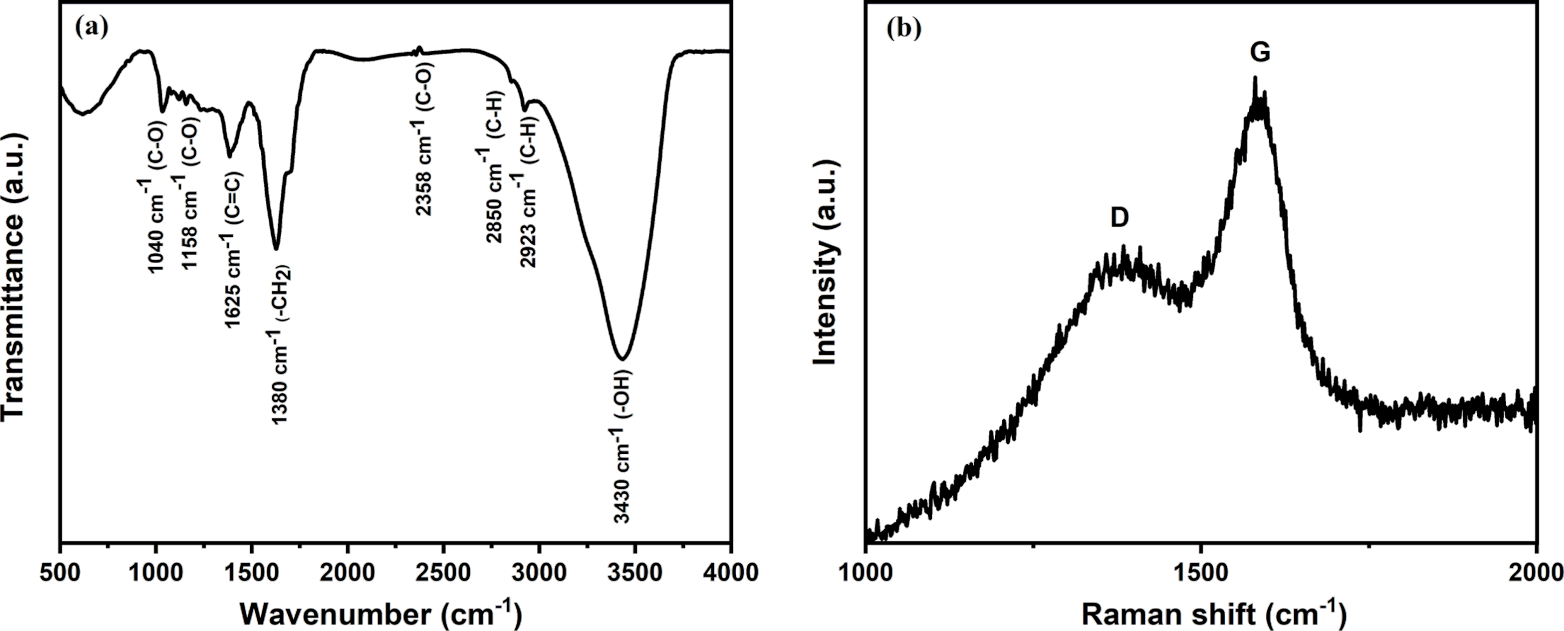
(a) FTIR spectrum and (b) Raman spectrum of GQDs.

Figure 5a shows the functional groups present on the surface of the GQDs measured using FTIR infrared spectroscopy. The broad absorption band at 3430 cm^−1^ corresponds to stretching vibrations of O–H bonds [36], which impart hydrophilicity to GQDs to form a dispersion in water. Similarly, the peaks at 2923 and 2850 cm^−1^ may be assigned to C–H stretching vibrations, the peaks at 2358, 1040, and 1158 cm^−1^ to C–O stretching vibrations, the peaks at 1625 cm^−1^ to C=C vibrations, and the peaks at 1380 cm^−1^ to C–H vibrations of alkyl groups [37]. It can be inferred that the surface of GQDs is passivated by surface groups that occur during the carbonization of glucose. The Raman spectrum of the GQDs in the spectral range of 1000–2000 cm^−1^ without any baseline correction displays typical D (ca. 1385 cm^−1^) and G bands (ca. 1585 cm^−1^) with an excitation wavelength of 532 nm as shown in Figure 5b, resembling those of a standard graphitic structure [38]. As a result of defects in the sp^2^-hybridized GQDs structure, the D band occurs due to transverse optical (TO) phonons about the *k* point of the Brillouin zone, while the G band arises from vibrations in rings of sp^2^-hybridized atoms inside the GQDs.

### Electrochemical studies

#### Electrochemical impedance spectroscopy

**Figure 6:**
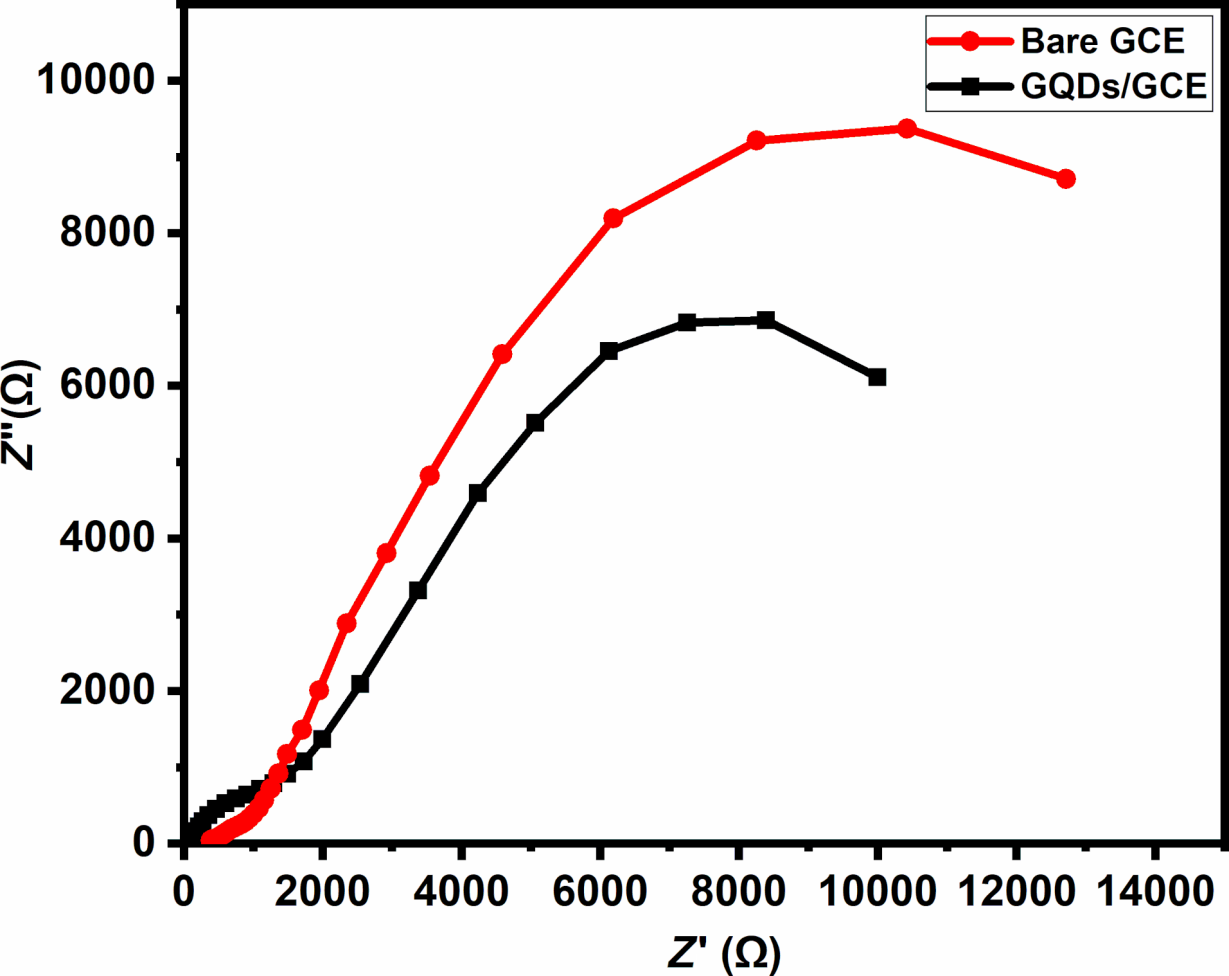
EIS measurement of 0.1 M KCl containing 0.05 M [Fe(CN)_6_]^3−/4−^ at the bare GCE and the GQDs/GCE working electrodes. *Z*′ and *Z*″ denote the real part and the imaginary part of the impedance, respectively.

In order to investigate the charge transfer on the electrode surfaces, electrochemical impedance spectroscopy (EIS) was used with the redox probe ferrocyanide. In EIS spectra, the semicircle component represents the charge transfer resistance (*R*_ct_) at the surface of the electrode. The Nyquist plots of the GQDs/GCE and the bare GCE are shown in Figure 6 in 0.1 M KCl solution containing 0.05 M [Fe(CN)_6_]^3−/4−^. The bare GCE electrode exhibits a semicircle with a resistance of about 12.71 kΩ. After modification with GQDs, the *R*_ct_ value decreases to about 9.98 kΩ. It can be inferred that, as a result of an increase in conductivity, K_3_Fe(CN)_6_ can reach the electrode surface more easily.

#### Cyclic voltammetry

**Figure 7:**
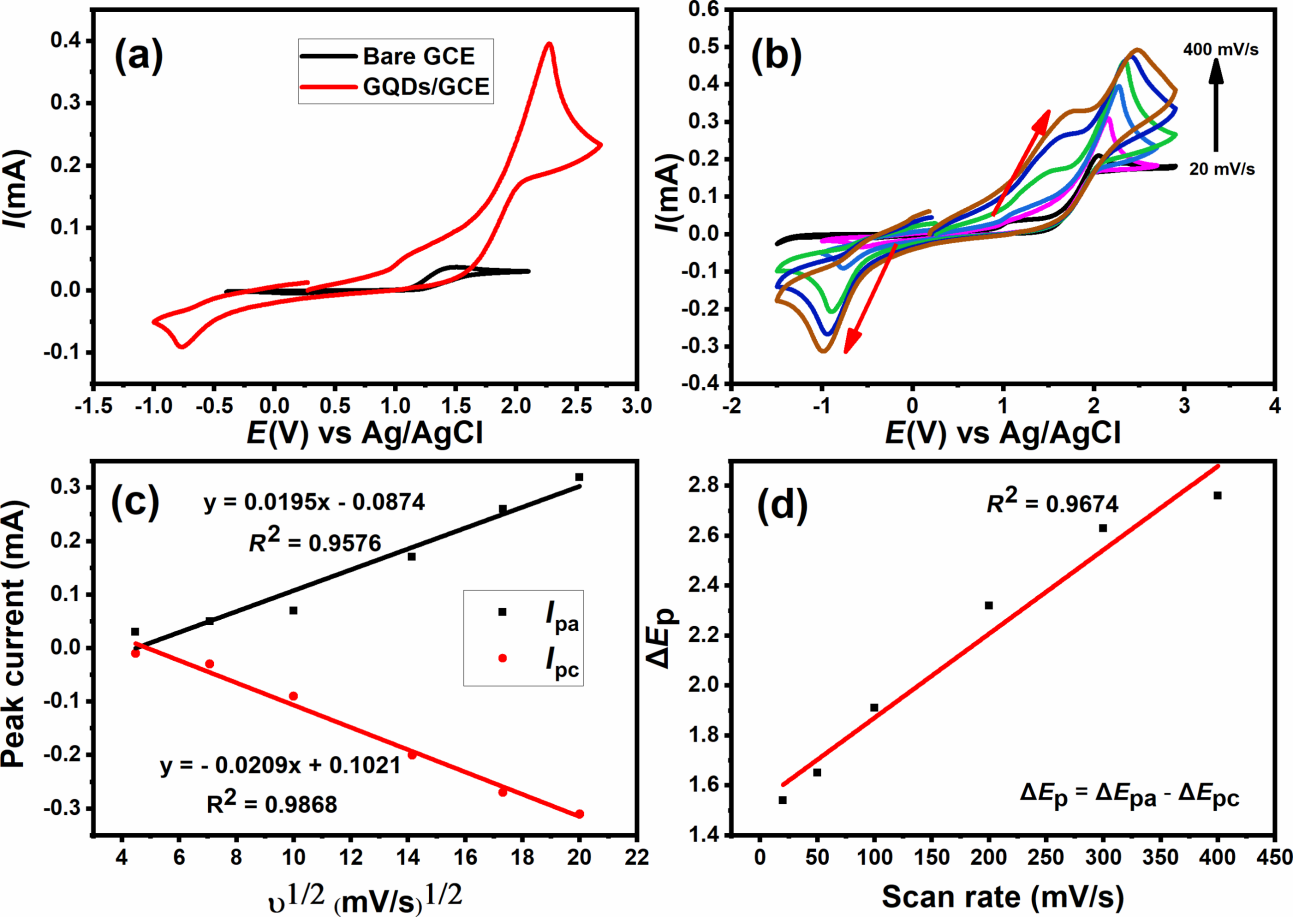
(a) Cyclic voltammograms of 0.1 M KCl containing 0.05 M [Fe(CN)_6_]^3−/4−^ at the bare GCE and GQDs/GCE electrodes (100 mV·s^−1^ scan rate), (b) cyclic voltammograms at the GQDs/GCE electrode at incremental scan rates from 20 to 400 mV·s^−1^, (c) plots of *I*_pa_ and *I*_pc_ as functions of υ^1/2^, and (d) plot of Δ*E*_p_ as function of the scan rate.

The redox electrochemical behavior of the bare GCE and GQDs/GCE electrodes was first evaluated using cyclic voltammetry in 0.1 M KCl containing 0.05 M [Fe(CN)_6_]^3−/4−^ at a scan rate of 100 mV·s^−1^, as shown in Figure 7a. The bare GCE exhibits well-defined anodic and cathodic redox peaks at 1.45 and 0.16 V, respectively. In the cathodic direction, GQDs/GCE exhibits a peak at −0.76 V, which can be assigned to the reduction of [Fe^III^(CN)_6_]^3−^ to [Fe^II^(CN)_6_]^4−^. In the anodic direction, GQDs/GCE exhibits two peaks, one at 1.14 V due to the oxidation of [Fe^II^(CN)_6_]^4−^ to [Fe^III^(CN)_6_]^3−^ and a second one at 2.27 V due to an oxidation of Fe^III^(CN)_6_]^3−^ to [Fe^IV^(CN)_6_]^2−^ as reported in [39]. As a result of the modification with GQDs, electron transfer was improved, resulting in a higher peak current and an electron-conducting channel on the modified electrode, showing an increase in peak current from 0.037 to 0.39 mA.

#### Effect of scan rate

Figure 7b shows cyclic voltammetry results of the GQDs/GCE electrode to study the interfacial kinetics from 20 mV·s^−1^ scan rate to 400 mV·s^−1^ scan rate. The increase in the square root of scan rates led to a linear increase in peak current for anodic and cathodic reactions, as shown in Figure 7c. For scan rates of 20 to 400 mV·s^−1^, an incremental scan rate results in a more positive anodic peak and a more negative cathodic peak, suggesting that the redox reaction is a reversible process. Moreover, Figure 7d shows that peak shift and scan rate have a linear relationship, indicating that electrochemical reactions at the electrode are diffusion-controlled, and the linear relationship (*R*^2^ = 0.9674) between peak height and scan rate suggests an enhanced electrochemical activity.

#### Electrochemical detection of malathion

**Figure 8:**
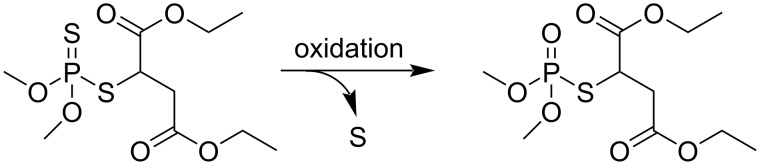
Oxidative desulfurization of malathion into malaoxon.

Using the modified working GQDs/GCE electrode as electrochemical sensor, a differential pulse voltammetry (DPV) analysis was conducted with various concentrations of malathion in 0.1 M PBS (pH 7) at a scan rate of 50 mV·s^−1^. Different concentrations of malathion were detected. The oxidative desulfurization of malathion into malaoxon (Figure 8) results in a current peak (centered at +1.9 V) at the GQDs/GCE electrode.

**Figure 9:**
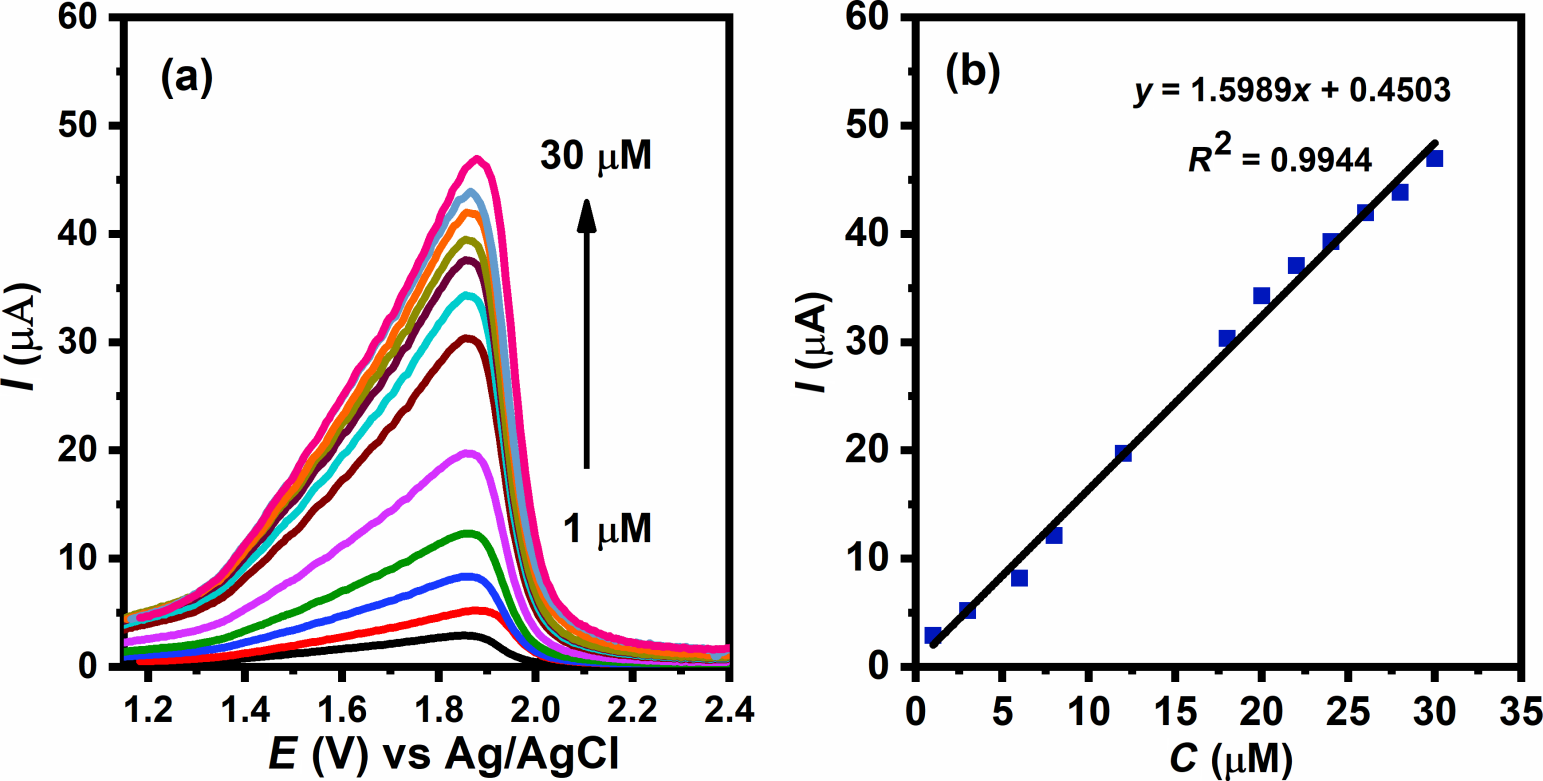
(a) Differential pulse voltammograms of diﬀerent concentrations of malathion (1 to 30 µM) at the GQDs/GCE electrode. (b) Peak current as function of the concentration of malathion.

The DPV study in Figure 9a shows an increase in the oxidation peak current as the concentration of malathion increases from 1 to 30 µM, suggesting that the GQDs/GCE electrode is sensitive towards malathion. As shown in Figure 9b, the linear regression equation of peak current and concentration for malathion detection is:


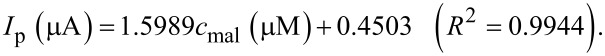


Using the equation *k*S_b_/*m* [35], where S_b_ represents standard deviation of the peak current of the blank, *m* represents the slope of the calibration plot, *k* has a value of 3, the limit of detection (LOD) was calculated to be 0.62 nM. In a similar manner, the limit of quantitation (LOQ) of 2.06 nM was calculated using a *k* value of 10. A comparison of the GQDs/GCE electrode with other existing electrodes for malathion detection is presented in Table 1. It indicates that the proposed GQDs-based electrode has a lower detection limit than other electrodes. The modified GQDs/GCE working electrode has an increased surface area to volume ratio due to the small size of GQDs. In addition, the different functional groups present on the GQDs surfaces provide additional active sites, which increase sensitivity and lower the limit of detection.

**Table 1:** Comparison of sensing parameters for the electrochemical detection of malathion using different electrode materials.^a^

Electrode	Technique	Limit of detection	Linear range	Reference

AChE-ZrO_2_/CHIT composite film/GCE	CV	1.3 × 10^−6^ M	1.0 × 10^−8^ to 5.9 × 10^−7^ M	[40]
AChE/PAn-PPy-MWCNTs/GCE	CV	1.0 ng·mL^−1^	0.01 to 0.5 μg·mL^−1^ and 1 to 25 μg·mL^−1^	[41]
Tyr/nano-Pt/graphene/GCE	chronoamperometry	3 ppb	5 to 30 ppb	[42]
PANI-ES/SWCNTs/graphite	DPV	2.0 × 10^−7^ M	(2.0 to 14) × 10^−7^ M	[43]
PLaE-CS/AuNPs-GNs/GCE	DPV	1.51 nM	1.5 to 1513.5 nM	[44]
poly(TTP)/AChE/GCE	CV	4.08 nM	9.99 to 99.01 nM	[45]
mitochondria-modified paper-based electrodes	CV	20 nM	20 to 60 nM	[46]
AuNP-CS-IL/PGE	SWV	0.68 nM	0.89 to 5.94 nM and 5.94 to 44.6 nM	[47]
CHIT-*g*-PANI	potentiometry	3.8 µM	2.0 to 62.5 µM	[48]
CuFe_2_O_4_-rGO/GCE	SWV	0.992 ± 0.007 ppm	0.5 to 8 ppm	[49]
FTO/PA6/PPy/CRGO	DPV	0.8 ng/mL	500 to 2 × 10^4^ ng·mL^−1^	[50]
GQDs/GCE	DPV	0.62 nM	1–30 µM	present work

^a^GCE: glassy carbon electrode, AChE: acetylcholinesterase, CHIT: chitosan, PAn: polyaniline, PPy: polypyrrole, MWCNTs: multi-walled carbon nanotubes, Tyr: tyrosinase, PANI-ES: polyaniline emiraldine salt, SWCNTs: single-walled carbon nanotubes, PLaE-CS: plant esterase–chitosan, AuNPs-GNs: gold nanoparticles–graphene nanosheets, poly(TTP): poly([2,2′:5′,2″]-terthiophene-3′-carbaldehyde, AuNP-CS-IL: gold nanoparticles–chitosan–ionic liquid, CHIT-*g*-PANI: chitosan-grafted polyaniline, rGO: reduced graphene oxide, FTO: fluorine tin oxide, PA6: polyamide 6, PPy: polypyrrole, CRGO: chemically reduced graphene oxide, CV: cyclic voltammetry, SWV: square wave voltammetry, DPV: differential pulse voltammetry.

#### Interference study

**Figure 10:**
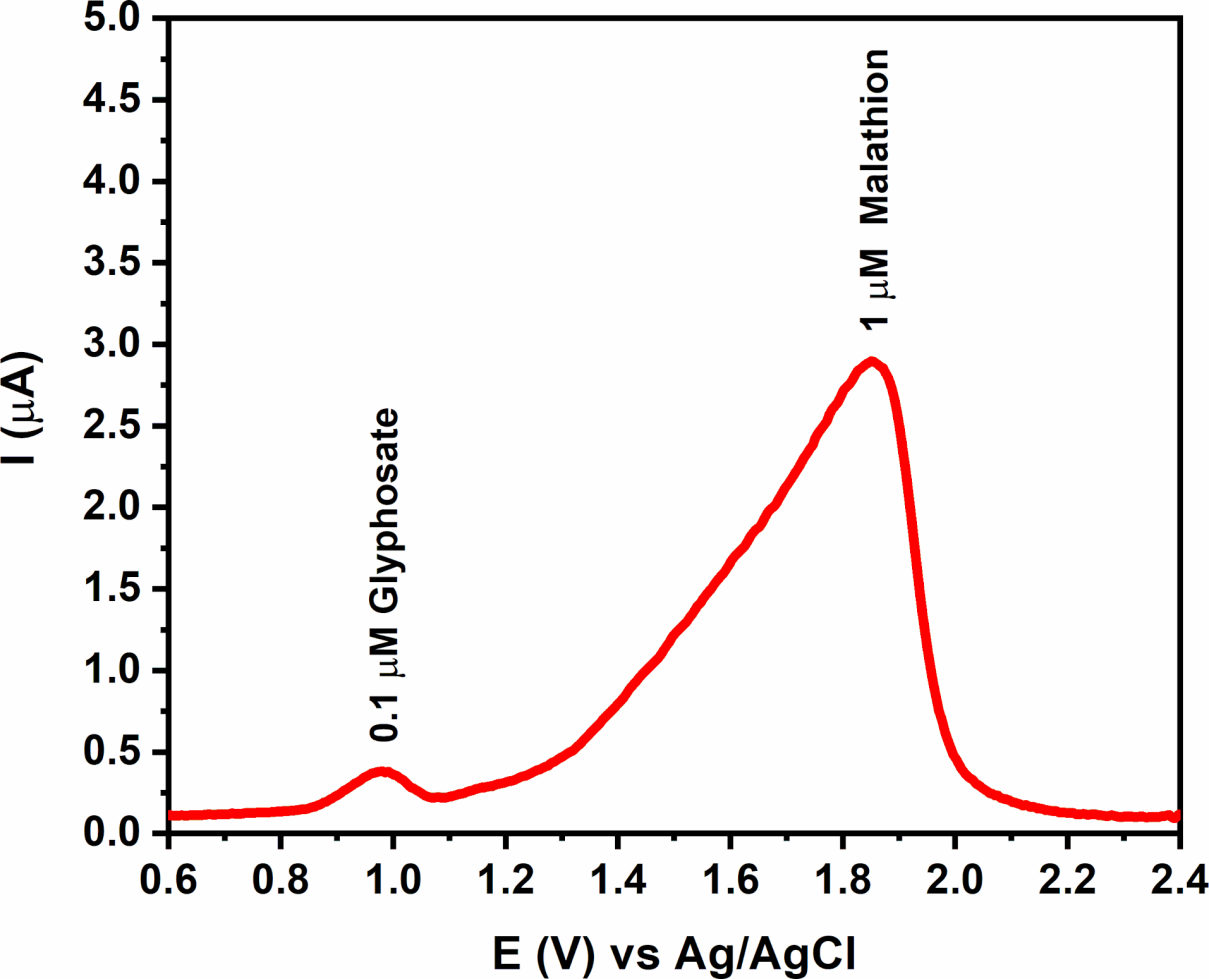
Differential pulse voltammogram of 1 µM malathion and 0.1 µM glyphosate using the GQDs/GCE working electrode.

The selectivity of the GQDs/GCE electrode towards malathion was examined by studying interfering effects in the presence of the organophosphate pesticide glyphosate. The DPV measurements showed that adding 0.1 µM glyphosate in the electrolytic solution containing 1 µM malathion shows no alteration in the peak potential for malathion detection (Figure 10).

## Conclusion

Graphene quantum dots (size range 5 to 40 nm) were chemically synthesized by using glucose as a precursor in a hydrothermal method. This paper describes the fabrication of an electrochemical nanosensor by modifying a bare glassy carbon electrode with GQDs. The oxidation–reduction behavior of the GQDs/GCE electrode was studied using cyclic voltammetry. Electrochemical impedance spectroscopy showed an increased charge transfer of the modified electrode. Differential pulse voltammetric analysis was performed to detect the organophosphate pesticide malathion. From 1 to 30 µM, a linear relationship was observed between the peak current and the malathion concentration. A detection limit of 0.62 nM was determined, and an interference study showed that the developed electrochemical sensor is selective for malathion. The sensor’s selectivity for malathion can be attributed to its surface composition of different functional groups providing specific sites for pesticide molecules. These results demonstrate that the GQDs/GCE electrochemical sensor is capable of detecting malathion over a wide linear range with low detection limits and high selectivity. The graphene-based nanosensor described here could be used in future to develop portable monitoring systems for water contamination.
